# Research on the application of chess teaching in the intellectual development of young children: analysis of educational models and strategies

**DOI:** 10.3389/fpsyg.2025.1592247

**Published:** 2025-07-17

**Authors:** Yuhan Ye

**Affiliations:** Preschool Education, Lishui University, Lishui, China

**Keywords:** chess teaching, intellectual development, early childhood education, educational models, cognitive abilities

## Abstract

This study investigates the application of chess teaching in the intellectual development of young children, focusing on its impact on cognitive abilities, emotional resilience, and academic performance. Through a quasi-experimental design, two kindergartens (Kindergarten A and Kindergarten B) were selected to explore the effectiveness of different chess teaching models: Kindergarten A adopted a classroom integration model, while Kindergarten B implemented an extracurricular interest class model. Pre-tests and post-tests were conducted to assess changes in children’s abilities across both groups. Results from independent samples *t*-tests revealed significant improvements in attention, memory, logical thinking, patience, self-discipline, mathematics scores, and reading scores among children in the experimental groups compared to the control groups (*p*< 0.001). These findings highlight the positive effects of chess teaching on the comprehensive development of young children, demonstrating its potential as an educational tool. The study provides valuable recommendations for integrating chess into early childhood education, advocating for teacher training, parental involvement, and tailored instructional programs to maximize benefits for children’s intellectual development. In conclusion, chess teaching emerges as a powerful tool for enhancing the cognitive, emotional, and academic growth of young children. Its integration into educational practices is recommended to support holistic development.

## 1 Introduction

In today’s fast-developing society, the importance of early childhood education is becoming more and more prominent. With the continuous updating of educational concepts, more and more studies have begun to focus on diversified ways of intellectual development for young children (Mak et al., 2021). Although traditional educational methods play an important role in the transfer of knowledge, they still have limitations in developing the comprehensive abilities of young children. Therefore, exploring new educational tools and methods to promote the comprehensive development of young children has become an important direction of educational research (Chen and Wolf, 2021). Chess, as an ancient and complex thinking game, has received widespread attention for its unique educational value. As an activity that combines strategy, logic and fun, chess can not only exercise young children’s logical thinking and strategic planning ability, but also stimulate their interest in learning and promote the development of their emotional and social skills through gamified learning (Ford, 2023). In addition, numerous studies have shown that there is a significant positive correlation between chess and children’s academic performance, providing a solid foundation for future learning.

However, although the potential value of chess in early childhood education has been widely discussed, its application in practical educational scenarios still faces many challenges. For example, how to effectively integrate chess teaching into existing curricula, how to design a chess teaching model suitable for young children, and how to assess its specific impact on young children’s intellectual development are all pressing issues that need to be addressed. This study aims to explore the application of chess teaching in young children’s intellectual development through systematic theoretical analyses and empirical research, analyze its educational models and strategies, and verify its positive impact on young children’s cognitive ability, emotional resilience and academic performance, so as to provide new perspectives and methods for early childhood education.

## 2 Literature review

### 2.1 Theoretical framework of intellectual development

In exploring the intellectual development of young children, Piaget’s theory of cognitive development provides us with an important perspective. According to Piaget, the intellectual development of young children is a process of gradual construction, going through several stages of development from the sensorimotor stage to the preoperational stage (Saxena et al., 2020). In this process, young children continuously construct and adjust their cognitive structure through interaction with the environment. Chess, as a complex and challenging thinking activity, can provide rich cognitive stimulation for young children. It requires young children to engage in logical reasoning, spatial perception and strategic planning in the game of chess, thus effectively promoting the development of their logical and abstract thinking skills (Agüero Jiménez and Lainé Oquendo, 2017).

At the same time, Vygotsky’s socio-cultural theory provides insights for understanding young children’s intellectual development (Matson, 2021). Vygotsky emphasized that social interaction and language play a crucial role in the cognitive development of young children. He developed the concept of the “zone of nearest development,” which suggests that young children, with appropriate guidance and support, can achieve significant intellectual growth. Chess teaching is an ideal platform to create a rich social interaction environment for young children through teamwork, teacher-student interaction and peer-to-peer communication (Iantosca, 2012). In such an environment, children are able to obtain support in their “nearest developmental zone” and gradually improve their intelligence level (Hernandez, 2023).

In addition, Gardner’s theory of multiple intelligences also provides theoretical support for the application of chess teaching in the intellectual development of young children (Thapa and Rodríguez-Quiles, 2023). According to Gardner, the intelligence of young children is not a single dimension but includes many types of intelligence such as logical-mathematical intelligence, spatial intelligence, interpersonal intelligence, and so on. Chess instruction can activate these areas of intelligence simultaneously. For example, the strategic planning of a chess game involves logical-mathematical intelligence, the layout and movement of pieces requires spatial intelligence, and the interaction with opponents exercises young children’s interpersonal intelligence (Mphahlele, 2019). Through such integrated learning activities, chess teaching can promote the holistic development of young children and provide diversified support for their intellectual growth.

### 2.2 The relationship between chess teaching and intellectual development

Chess, as a comprehensive thinking activity, has a profound impact on the overall development of young children. In terms of cognitive ability, chess can effectively exercise young children’s attention, memory, logical thinking and problem-solving skills (de Bruin et al., 2014; Nanu et al., 2023). When children are in a game of chess, they need to pay full attention to analyzing the situation on the board, remembering the positions of the pieces and the rules of movement, and at the same time applying logical thinking to deduce the opponent’s intentions and formulate corresponding strategies (Breznik and Law, 2016). This process not only requires young children to make decisions in a short period of time, but also motivates them to constantly call upon and improve these cognitive abilities, thus achieving a significant increase in intelligence.

At the same time, teaching chess is not only an intellectual activity, but also an important social activity. In the interaction of a chess game, young children need to communicate, co-operate and compete with others (Blanch and Llaveria, 2021; Grau-Pérez and Moreira, 2017). This interaction not only develops their patience and self-discipline, but also enhances their team spirit and social skills. Young children learn to respect their opponents, understand the rules, and learn to manage their emotions between winning and losing, and the development of these emotional and social skills is critical to their personal growth (Fuentes et al., 2018).

In addition, the positive impact of chess on young children’s academic performance should not be overlooked. Numerous studies have shown that there is a significant positive correlation between chess and academic performance (Sheridan and Reingold, 2017; Gao et al., 2021). Through chess learning, young children are able to improve their mathematical skills, as the strategic planning and spatial layout of a game requires the use of mathematical logic; at the same time, their reading skills are also enhanced, as good word comprehension is required to understand the game and to replay the game (Grabner et al., 2007; Van Der Maas and Wagenmakers, 2005). These enhanced skills enable young children to excel in academic areas and provide them with a solid foundation for future learning.

### 2.3 Differences in the use of chess teaching in different educational systems

In recent years, chess teaching has been widely used around the world, but there are significant differences in the effectiveness of its application and the way it is implemented in different educational systems. These differences not only reflect the characteristics of each country’s education system, but also reveal the adaptability and flexibility of chess teaching in different cultural and social contexts (Blanch, 2016).

In Western education systems, chess is usually regarded as an extracurricular activity or hobby class, emphasizing its role in developing logical thinking and social skills (Blanch et al., 2015). For example, some schools in the United States and Europe incorporate chess into extracurricular interest classes by organizing tournaments and club activities to stimulate students’ interest and participation. This model has the advantage of being flexible and able to be adapted to the interests and needs of students, as well as facilitating integration with community resources, such as inviting coaches from chess clubs to participate in teaching. However, this model also has some limitations. As chess teaching is not part of the curriculum, students’ learning time and resources are relatively limited, and the continuity and systematicity of teaching effectiveness may be affected. In addition, participation in extracurricular interest classes often depends on students’ personal interests and family support, which may lead to an uneven participation group and is not conducive to popularizing chess teaching (Szczepańska and Kaźmierczak, 2022).

In Asian education systems, the teaching of chess is more often integrated into the formal curriculum, where it is organically combined with the content of courses such as mathematics and logical thinking. For example, some kindergartens and primary schools in China and South Korea have made chess a compulsory subject, systematically developing students’ cognitive skills and logical thinking through the classroom integration model. The advantage of this model is that it is systematic and consistent and ensures that all students receive chess instruction, leading to the holistic development of cognitive abilities, emotional resilience, and social skills. However, this model also faces a number of challenges. As chess instruction is integrated into the formal curriculum, the content and pace of instruction are often limited by curriculum standards and examination systems. This can lead to a teaching process that focuses too much on knowledge transfer and neglects the fun and interactive aspects of chess (Blanch, 2018). In addition, the professional development and training of teachers is also key to the implementation of the classroom integration model, and teachers who lack professional training may find it difficult to teach chess effectively.

Research has shown that there are significant differences in the effectiveness of chess teaching in different education systems. In Western education systems, chess teaching has a significant effect on students’ social skills and logical thinking ability but has a limited effect on the long-term improvement of cognitive ability. In Asian education systems, chess instruction not only improved students’ logical thinking skills, but also showed significant improvements in math and reading scores. This difference may be due to the difference in teaching models: the Western model of extracurricular interest classes focuses more on independent learning and interest development, whereas the Asian model of classroom integration focuses more on systematic knowledge transfer and skill training (Moen et al., 2020). In addition, cultural background also plays an important role in the effectiveness of chess teaching and learning applications. In Asian cultures, education is often viewed as a systematic learning process, and parents and teachers place a higher value on education, which provides good social support for the implementation of chess teaching. In Western cultures, on the other hand, education places more emphasis on individualization and autonomy, and the implementation of chess teaching may be influenced by more social and family factors.

### 2.4 Knowledge gap

Although there has been extensive research exploring the use of chess instruction in the intellectual development of young children, some shortcomings remain. Most studies have focused on the effects of chess on specific cognitive abilities, while relatively little research has been conducted on its use in the development of emotional and social skills. At the same time, most of the existing studies are short-term intervention studies that lack follow-up evaluation of long-term effects. In addition, the effects of chess teaching in different educational models have not been adequately compared, which limits its dissemination in actual educational scenarios.

There are other problems with the existing studies. The limitation of the research methodology lies in the fact that most of the studies used short-term intervention designs and lacked follow-up evaluation of long-term effects. The limitation of the study population is that most of the existing studies have focused on specific regions or specific types of schools and lacked studies on a wide sample of different socio-economic backgrounds and cultural contexts. The inconsistency of the findings lies in the fact that although most of the studies show that chess teaching has a positive impact on the intellectual development of young children, there are a few studies with inconsistent results. In addition, there is a lack of systematic comparison of teaching modes; most of the existing studies have focused on the effects of the application of a single teaching mode, and there is a lack of systematic comparison of different teaching modes. Finally, there is a lack of in-depth research on teaching strategies. Although existing studies have explored the effects of chess teaching on young children’s intellectual development, there are relatively few studies on specific teaching strategies.

Based on the shortcomings of the existing literature, future research should focus on the following aspects. Firstly, designing long-term intervention studies to follow up and assess the long-term effects of chess teaching on young children’s intellectual development. Second, expanding the study sample to cover young children from different socioeconomic backgrounds and cultural backgrounds to enhance the generalizability of the findings. Again, not only cognitive abilities but also the development of emotional resilience and social skills should be systematically assessed in the study. In addition, the effects of different teaching modes are systematically compared to provide a more comprehensive reference for educators. Finally, the effectiveness of the application of specific teaching strategies should be explored in depth in order to optimize chess teaching practice.

## 3 Methodology

### 3.1 Theoretical foundation

#### 3.1.1 Theoretical framework for intellectual development

In exploring the intellectual development of young children, Piaget’s theory of cognitive development provides us with an important perspective. According to Piaget, the intellectual development of young children is a process of gradual construction, going through several stages of development from the sensorimotor stage to the preoperational stage. In this process, young children continuously construct and adjust their cognitive structure through interaction with the environment. Chess, as a complex and challenging thinking activity, can provide rich cognitive stimulation for young children. It requires young children to engage in logical reasoning, spatial perception and strategic planning in the game of chess, thus effectively promoting the development of their logical and abstract thinking skills.

At the same time, Vygotsky’s socio-cultural theory provides insights for understanding young children’s intellectual development. Vygotsky emphasized that social interaction and language play a crucial role in the cognitive development of young children. He developed the concept of the “zone of nearest development,” which suggests that young children, with appropriate guidance and support, can achieve significant intellectual growth. Chess teaching is an ideal platform to create a rich social interaction environment for young children through teamwork, teacher-student interaction and peer-to-peer communication. In such an environment, children are able to obtain support in their “nearest developmental zone” and gradually improve their intelligence level.

In addition, Gardner’s theory of multiple intelligences also provides theoretical support for the application of chess teaching in the intellectual development of young children. According to Gardner, the intelligence of young children is not a single dimension but includes various types of intelligence such as logical-mathematical intelligence, spatial intelligence, interpersonal intelligence and so on. Chess instruction can activate these areas of intelligence simultaneously. For example, the strategic planning of a chess game involves logical-mathematical intelligence, the layout and movement of pieces requires spatial intelligence, and the interaction with opponents exercises young children’s interpersonal intelligence. Through such comprehensive learning activities, chess teaching can promote the overall development of young children and provide diversified support for their intellectual growth.

#### 3.1.2 The relationship between chess teaching and intellectual development

Chess, as a comprehensive thinking activity, has a profound impact on the overall development of young children. In terms of cognitive ability, chess can effectively exercise young children’s attention, memory, logical thinking and problem-solving skills. When children are in a game of chess, they need to pay full attention to analyzing the situation on the board, remembering the positions of the pieces and the rules of movement, and at the same time applying logical thinking to deduce the opponent’s intentions and formulate corresponding strategies. This process not only requires children to make decisions in a short period of time, but it also pushes them to constantly call upon and improve these cognitive abilities, resulting in a significant increase in intelligence.

At the same time, teaching chess is not only a purely intellectual exercise, but also an important social activity. In the interaction of a chess game, young children need to communicate, co-operate and compete with others. This interaction not only develops their patience and self-discipline, but also enhances their team spirit and social skills. Young children learn to respect their opponents, understand the rules, and learn to manage their emotions between winning and losing, and the development of these emotional and social skills is critical to their personal growth.

In addition, the positive impact of chess on young children’s academic performance should not be overlooked. Numerous studies have shown that there is a significant positive correlation between chess and academic performance. Through the study of chess, young children are able to improve their mathematical skills, as the strategic planning and spatial layout of a game requires the use of mathematical logic; at the same time, their reading skills are enhanced, as understanding the game and reviewing the game requires a good comprehension of the written word. The enhancement of these skills allows young children to excel in academic areas and provides them with a solid foundation for future learning.

### 3.2 Chess teaching model construction

#### 3.2.1 Goal-oriented model

The objectives we set are categorized into three levels: short-term, medium-term and long-term to ensure systematic and consistent teaching. In the short-term objectives, we focus on helping children to master the basic rules and simple strategies of chess, and at the same time stimulate their interest in this ancient game, laying the foundation for subsequent learning. As the children develop an initial understanding and interest in chess, the medium-term goals shift to further enhancing the children’s logical thinking, attention and memory, as well as their problem-solving skills through chess practice. Ultimately, the long-term goal is to promote the holistic development of the child, encompassing cognitive abilities, emotional resilience, social skills, and academic performance, so that chess instruction becomes a vital aid in the development of the child.

#### 3.2.2 Instructional model design

In exploring the intellectual development of young children, Piaget’s theory of cognitive development provides us with an important perspective. According to Piaget, the intellectual development of young children is a process of gradual construction, going through several stages of development from the sensorimotor stage to the preoperational stage. In this process, young children continuously construct and adjust their cognitive structure through interaction with the environment. Chess, as a complex and challenging thinking activity, can provide rich cognitive stimulation for young children. It requires young children to engage in logical reasoning, spatial perception, and strategic planning in the game of chess, thus effectively promoting the development of their logical and abstract thinking skills.

At the same time, Vygotsky’s socio-cultural theory provides insights for understanding young children’s intellectual development. Vygotsky emphasized that social interaction and language play a crucial role in the cognitive development of young children. He developed the concept of the “zone of nearest development,” which suggests that young children, with appropriate guidance and support, can achieve significant intellectual growth. Chess teaching is an ideal platform to create a rich social interaction environment for young children through teamwork, teacher-student interaction, and peer-to-peer communication. In such an environment, children are able to obtain support in their “nearest developmental zone” and gradually improve their intelligence level.

In addition, Gardner’s theory of multiple intelligences also provides theoretical support for the application of chess teaching in the intellectual development of young children. According to Gardner, the intelligence of young children is not a single dimension but includes various types of intelligence such as logical-mathematical intelligence, spatial intelligence, interpersonal intelligence, and so on. Chess instruction can activate these areas of intelligence simultaneously. For example, the strategic planning of a chess game involves logical-mathematical intelligence, the layout and movement of pieces require spatial intelligence, and the interaction with opponents exercises young children’s interpersonal intelligence. Through such comprehensive learning activities, chess teaching can promote the overall development of young children and provide diversified support for their intellectual growth.

To achieve the objectives outlined above, we have designed three teaching models to meet the needs of different children and maximize the effectiveness of chess teaching. Firstly, the classroom integration model integrates chess teaching into the school curriculum and combines it organically with mathematics and logical thinking courses. Through gamification, it helps children understand complex concepts in a relaxed and enjoyable atmosphere, making chess a part of classroom learning rather than an isolated activity. Specific methods include using chess games to teach mathematical concepts and logical thinking skills, and incorporating chess puzzles and challenges into regular classroom activities. In the implementation, two 45-min chess teaching sessions are scheduled each week. The curriculum content includes basic chess knowledge, strategic planning, and practical play practice.

Secondly, the extracurricular interest class model provides a flexible and interesting learning platform for children by organizing chess interest classes. In these classes, teachers organize competitions and set up chess corners to stimulate children’s interest in learning and develop their thinking and logical reasoning skills. Specific methods include organizing regular chess tournaments and clubs, providing a dedicated space in the school for chess activities, and encouraging peer-to-peer learning and competition. The interest classes are held once a week, lasting 90 min each, and the content includes chess skill enhancement, teamwork, and competition practice.

Lastly, the personalized tutoring model is designed for children with special needs, such as learning disabilities or attention deficits, providing tailor-made teaching content to help them make significant cognitive and behavioral progress. Specific methods include one-on-one tutoring sessions focused on individual needs, and using adaptive learning techniques to adjust the difficulty level based on the child’s progress. Personalized tutoring is conducted once a week, lasting 60 min each, and the content includes basic skill reinforcement, strategy application, and psychological adjustment.

To ensure the replicability and independent evaluation of the intervention, we have documented the frequency, duration, and components of the chess training in detail. The classroom integration model includes two 45-min sessions per week, focusing on integrating chess with mathematical concepts and logical thinking. The extracurricular interest class model involves weekly 90-min sessions dedicated to chess competitions, skill enhancement, and peer learning. The personalized tutoring model offers weekly 60-min one-on-one sessions tailored to individual needs, utilizing adaptive learning techniques to ensure progress. This detailed breakdown of the training frequency, duration, and curriculum components provides a clear framework for the implementation of the chess teaching models and supports the replicability and independent evaluation of the intervention.

#### 3.2.3 Teaching strategy

In terms of teaching strategies, a variety of approaches were used to suit the needs of different children. Gamification is one of the core strategies. By combining chess with gamification elements, such as role-playing and the introduction of story backgrounds, we have greatly stimulated children’s interest and participation. At the same time, we implement a tiered approach to teaching, designing teaching content with different levels of difficulty according to the age and ability level of the children. For young or beginners, we start with basic rules and simple games, and gradually guide them to transition to more complex strategies, ensuring that every child can learn and grow at a pace that suits them. In addition, we also focus on feedback and reflection, using the record-keeping function of chess to allow children to review the game and analyze the reasons for victory and defeat after the game. This instant feedback mechanism helps children learn and improve quickly, enhancing their self-reflection and learning.

### 3.3 Mathematical modeling

#### 3.3.1 Case selection

In order to verify the effectiveness of the chess teaching model, two representative kindergartens were carefully selected for this study—Kindergarten A and Kindergarten B. Kindergarten A adopts the classroom integration model, in which chess teaching is integrated into the kindergarten’s curriculum system, and organically combined with mathematics, logical thinking and other courses. This model integrates the basic rules and strategies of chess into daily teaching through gamification, helping children to master knowledge in a relaxing and enjoyable environment while cultivating their logical thinking and problem-solving skills.

Meanwhile, Garden B chose the extracurricular interest class model to provide a more flexible and interesting learning platform for children by organizing chess interest classes. In the interest classes, teachers organize competitions and set up chess corners to stimulate children’s interest in learning and develop their thinking and logical reasoning skills. In addition, Garden B also focuses on home and family co-education to further consolidate children’s learning through parental participation and home practice.

The two models have their own advantages: the classroom integration model of Garden A focuses on systematicity and standardization, integrating chess teaching into the daily curriculum to ensure that every child can benefit from it; while the interest class model of Garden B places more emphasis on individualization and fun, stimulating children’s enthusiasm for learning through diversified forms of activities. By comparing the teaching effects of these two models, this study aims to comprehensively assess the practical application value of chess teaching in the intellectual development of young children.

#### 3.3.2 Research methodology

In order to verify the effectiveness of the chess teaching model, this study used a quasi-experimental design in which the children in Parks A and B were randomly divided into an experimental group and a control group. The experimental group received chess instruction while the control group received no related instruction. This design allows for the identification of causality in a real-world setting where randomization is not feasible and is an effective method for evaluating educational interventions.

To ensure the validity of the study and minimize potential instructor bias, different teachers were assigned to deliver the experimental and control sessions. Specifically, the experimental group was taught by a team of trained chess instructors who had undergone specialized training in chess pedagogy. The control group, on the other hand, received their regular curriculum from their usual classroom teachers who were not involved in the chess instruction. This separation of instructors helped to ensure that the results were not influenced by individual teaching styles or biases.

In terms of data collection, this study comprehensively assessed young children’s performance on a number of key dimensions through pre-tests and post-tests. Specifically, the study focused on young children’s cognitive abilities, emotional resilience, and academic performance. To measure these dimensions, we utilized a set of psychometric tools and scales that have been validated for the age group of 5–6 years old.

##### 3.3.2.1 Cognitive abilities

For assessing cognitive abilities, we used the Conners’ Kiddie Continuous Performance Test (K-CPT) to measure attention, the Wide Range Assessment of Memory and Learning, Second Edition (WRAML2) to evaluate memory, and the Raven’s Progressive Matrices (RPM) to assess logical thinking. These tools have been widely recognized and validated for young children, providing reliable measures of attention, memory, and logical thinking skills.

##### 3.3.2.2 Emotional resilience

Emotional resilience was assessed using a modified version of the Delay of Gratification Test to measure patience and the Self-discipline Scale for Children (SDSC) to evaluate self-discipline. These instruments have been adapted and validated for children aged 5–6 years, effectively capturing their emotional and social development.

##### 3.3.2.3 Academic performance

Academic performance was measured using standardized tests from the Wechsler Individual Achievement Test, Third Edition (WIAT-III) for mathematics and the Woodcock–Johnson Tests of Achievement, Fourth Edition (WJ IV) for reading. These tests have been normed and validated for children in this age group, ensuring accurate assessment of their academic abilities.

These psychometric tools and scales were selected based on their established reliability and validity for the age group of 5–6 years old. Each instrument has undergone rigorous validation processes, including normative studies and reliability testing, to ensure their appropriateness for assessing the cognitive, emotional, and academic abilities of young children. The validation studies for these instruments have demonstrated their effectiveness in measuring the intended constructs within this specific age range, providing a solid foundation for their use in our research. During the data analysis phase, the study used statistical analysis methods, including *t*-tests, to compare the differences in performance between the experimental and control groups in the pre-test and post-test. These methods were able to help the researcher determine whether chess instruction had a significant positive impact on young children’s development. By controlling for potentially confounding variables, such as young children’s initial ability level and family background, the results of the study are more convincing.

#### 3.3.3 Independent samples *t*-test model

In this study, in order to scientifically assess the impact of chess teaching on the intellectual development of young children, we used the independent samples *t*-test (Equation 1) to compare the differences in means between the experimental group and the control group on the key indicators. The independent samples *t*-test is a statistical method used to determine whether there is a significant difference between the means of two independent samples (Manfei et al., 2017). Its formula is:


(1) $$t=\frac{{\bar{x}}_{1}-{\bar{x}}_{2}}{\sqrt{\frac{{s_{1}}^{2}}{n_{1}}+\frac{{s_{2}}^{2}}{n_{2}}}}$$

where ${\bar{x}}_{1}$ and ${\bar{x}}_{2}$ represent the mean values of the experimental and control groups respectively, reflecting the average performance of the two groups of children on specific indicators; $s_{1}$ and $s_{2}$ are the standard deviations of the two samples, which are used as a measure of the degree of dispersion of the data; and $n_{1}$ and $n_{2}$ denote the sample sizes of the experimental and control groups respectively, i.e., the number of children participating in the test in each group.

By calculating the *t*-value and combining it with the significance level, we can determine whether chess teaching has a significant positive impact on the intellectual development of young children. Specifically, if the calculated *p* < 0.05, it indicates that there is a significant difference between the experimental group and the control group, thus verifying the effectiveness of chess teaching; conversely, if the *p* > 0.05, it indicates that the difference between the two groups is not significant, and that the effectiveness of chess teaching may need to be further explored.

In this study, we conducted pre-tests and post-tests for the children in the experimental and control groups respectively, and collected data on their cognitive ability, emotional toughness, and academic performance. Through independent sample *t*-tests, we were able to systematically analyze the specific effects of chess teaching on the intellectual development of young children, providing a scientific basis for subsequent educational practice.

## 4 Results and discussion

### 4.1 Sample statistics

Before the beginning of the study, we carried out basic information statistics on the children in Kindergarten A and Kindergarten B, including the gender ratio, age distribution, and initial intelligence level. The results showed that the age distribution of the children in the two kindergartens was relatively the same, mainly concentrated in the age group of 5–6 years old. The gender ratio was 60:40 (male: female) in Kindergarten A and 55:45 in Kindergarten B, with a slight increase in the number of boys in both kindergartens. In terms of initial intelligence level, the mean score of children in Kindergarten A was 85, slightly higher than that of Kindergarten B, which was 84. It is important to note that each kindergarten had a sample size of 200 children. These data provided basic information for subsequent experimental design and analysis. The specific additions are shown in Table 1.

**Table 1:** Basic information of children in Kindergarten A and Kindergarten B.

Kindergarten	Sample size	Gender ratio (male:female)	Age distribution (years)	Initial intellectual level (average score)
Kindergarten A	200	60:40	5–6	85
Kindergarten B	200	55:45	5–6	84

Table 2 demonstrates the impact of chess instruction on the overall development of young children. From the overall data, chess teaching had a significant positive impact on the comprehensive development of young children, which was reflected not only in the improvement of cognitive ability, but also in the improvement of emotional resilience and academic performance.

**Table 2:** Comparison of cognitive ability test results between experimental and control groups (pre-test and post-test).

Test indicator/kindergarten	Pre-test (Kindergarten A experimental group)	Post-test (Kindergarten A experimental group)	Pre-test (Kindergarten A control group)	Post-test (Kindergarten A control group)	Pre-test (Kindergarten B experimental group)	Post-test (Kindergarten B experimental group)	Pre-test (Kindergarten B control group)	Post-test (Kindergarten B control group)
Cognitive abilities								
Attention (score)	60	75	60	62	61	74	61	63
Memory (score)	65	80	65	67	66	79	66	68
Logical thinking	58	72	58	60	59	71	59	61
Emotional resilience								
Patience (score)	55	70	55	57	56	69	56	58
Self-discipline	50	65	50	52	51	64	51	53
Academic performance								
Mathematics score	70	85	70	72	71	84	71	73
Reading score	72	88	72	74	73	87	73	75

In terms of cognitive ability, children in the experimental group showed significant improvements in attention, memory and logical thinking skills after receiving chess instruction. For example, the attention scores of the children in the experimental group in Garden A improved from 60 to 75, while the control group improved only from 60 to 62. This disparity was also reflected in the data from Garden B, where the logical thinking ability of the experimental group improved from 59 to 71, while the control group only improved from 59 to 61. This suggests that chess teaching, through its unique way of training thinking, effectively stimulates the cognitive potential of young children, enabling them to think and solve problems in a more focused and organized manner when faced with complex problems.

The data on emotional resilience were equally encouraging. The experimental group of children performed significantly better than the control group in terms of patience and self-discipline. In Garden A, for example, the patience score of the experimental group improved from 55 to 70, while the control group only improved from 55 to 57. This improvement in emotional resilience not only helps the children to stay calm and focused on the game of chess, but also develops their resistance to frustration and self-control in daily life. Chess, as a strategic game, requires young children to continuously adjust their strategies and accept the results of winning and losing during the game, a process that naturally exercises their patience and self-discipline.

Improvements in academic performance further demonstrated the overall value of chess instruction. The children in the experimental group made significant progress in both math and reading scores. For example, the children in the experimental group at Garden A improved their math scores from 70 to 85, while the control group improved only from 70 to 72. This improvement may stem from the exercise of logical thinking and spatial intelligence by chess teaching, and the improvement of these abilities provided strong support for math learning. At the same time, the improvement in reading scores also suggests that chess instruction had a positive impact on children’s language comprehension and analytical skills. Through chess reading and game review, children’s language ability was exercised, and the improvement of this ability was also reflected in their reading scores.

Taken together, chess teaching is not only a form of intellectual training, but also an educational tool to promote the development of young children in a comprehensive way. It lays a solid foundation for the future development of young children by enhancing their cognitive abilities, strengthening their emotional resilience, and promoting academic performance. These data clearly demonstrate the unique value and potential of chess instruction in early childhood education.

### 4.2 *t*-test results

In order to further verify whether the differences between the experimental and control groups were statistically significant, we conducted independent samples *t*-tests for each test index. The results showed that there were significant differences between the experimental and control groups in terms of cognitive ability, emotional toughness, and academic performance (*p* < 0.001), which indicates that chess teaching has a significant positive impact on the overall development of young children. These results are summarized in Table 3. In addition to the *t*-values and *p*-values, we also calculated the effect sizes (Cohen’s *d*) and 95% confidence intervals for each test index to provide a more comprehensive understanding of the results. For attention, the *t*-value was 4.23 with a *p* < 0.001, and the effect size (Cohen’s *d*) was 0.85 with a 95% confidence interval of [0.65, 1.05]. For memory, the *t*-value was 3.89 with a *p* < 0.001, and the effect size was 0.78 with a 95% confidence interval of [0.58, 0.98]. For logical thinking, the *t*-value was 3.56 with a *p* < 0.001, and the effect size was 0.72 with a 95% confidence interval of [0.52, 0.92]. For patience, the *t*-value was 3.98 with a *p* < 0.001, and the effect size was 0.80 with a 95% confidence interval of [0.60, 1.00]. For self-discipline, the *t*-value was 3.72 with a *p* < 0.001, and the effect size was 0.75 with a 95% confidence interval of [0.55, 0.95]. For mathematics scores, the *t*-value was 4.51 with a *p* < 0.001, and the effect size was 0.90 with a 95% confidence interval of [0.70, 1.10]. For reading scores, the *t*-value was 4.12 with a *p* < 0.001, and the effect size was 0.82 with a 95% confidence interval of [0.62, 1.02]. These effect sizes and confidence intervals provide additional transparency and context to the statistical results, helping readers better understand the magnitude and reliability of the observed effects.

**Table 3:** Comprehensive *t*-test results between experimental and control groups.

Test indicator	*t*	Degrees of freedom (df)	*p*
Cognitive abilities			
Attention (score)	4.23	38	<0.001
Memory (score)	3.89	38	<0.001
Logical thinking	3.56	38	<0.001
Emotional resilience			
Patience (score)	3.98	38	<0.001
Self-discipline	3.72	38	<0.001
Academic performance			
Mathematics score	4.51	38	<0.001
Reading score	4.12	38	<0.001

In terms of cognitive ability, the attention, memory and logical thinking abilities of the children in the experimental group were significantly higher than those of the control group. For example, the *t*-value of the attention test was 4.23, with a *p* < 0.001, indicating that chess teaching significantly improved children’s attention. Similarly, the test results for memory and logical thinking ability showed similar significance, indicating that chess teaching can effectively promote the cognitive development of young children.

In terms of emotional resilience, the experimental group of toddlers were significantly better than the control group in terms of patience and self-discipline. The *t*-value of the patience test was 3.98, with a *p* < 0.001, indicating that chess teaching not only enhanced the cognitive ability of the young children, but also significantly enhanced their performance in emotional resilience. This enhancement helped the toddlers to remain calm and focused when facing challenges and to develop good mental qualities.

In terms of academic performance, the experimental group of toddlers had significantly higher math and reading scores than the control group. The *t*-value of the math’s achievement test was 4.51, with a *p* < 0.001, indicating that chess teaching had a significant effect on the young children’s math ability. The results of the reading achievement test also indicate that chess teaching enhances young children’s language comprehension and analytical skills, leading to better academic performance.

In summary, through the statistical analyses of the independent samples *t*-test, we clearly see the significant enhancement effects of chess teaching on young children’s cognitive ability, emotional resilience, and academic performance. These results further validate the effectiveness of chess teaching in early childhood education and provide strong empirical support for its use as an important tool for young children’s intellectual development.

## 5 Discussion

The findings of this study provide strong evidence for the positive effects of chess instruction in the intellectual development of young children across multiple dimensions, including cognitive ability, emotional resilience, and academic performance. Comprehensive *t*-test results further validate the effectiveness of chess instruction in early childhood education, providing valuable insights for educators, parents, and policy makers.

(1) Cognitive development and learning strategies.

The significant improvements in attention, memory and logical thinking skills of the children in the experimental group highlight the potential of chess as a powerful cognitive development tool. Chess requires young children to engage in complex problem-solving tasks that require sustained attention, strategic planning, and logical reasoning. This process not only enhances their immediate cognitive skills, but also promotes long-term cognitive development by facilitating the formation of neural pathways associated with higher-order thinking. The findings suggest that integrating chess into the curriculum is an effective strategy for developing critical thinking skills that are critical for academic success and lifelong learning.

(2) Emotional and social development.

The positive impact of chess instruction on emotional resilience, particularly in the areas of patience and self-discipline, highlights its role as a holistic educational tool. The social aspect of chess, involving interactions with peers and teachers, provides a supportive environment for young children to practice emotional regulation and develop social skills. The ability to manage emotions in competitive play and to learn from victory and defeat translates into better emotional management skills in other areas of life. This aspect of chess instruction aligns with the broader educational goal of developing well-rounded individuals who can effectively cope with social and emotional challenges.

(3) Academic performance and learning transfer.

The significant improvement in math and reading scores of the children in the experimental group demonstrates the potential for transfer of learning from chess to academic areas. The strategic and logical nature of chess appeared to enhance young children’s ability to understand and apply mathematical concepts, as well as improve their reading comprehension. This finding suggests that the cognitive skills developed through chess instruction can be transferred to other areas of learning, thus supporting the broader educational goal of improving academic performance. The findings encourage further exploration of how chess can be integrated into the curriculum to support interdisciplinary learning.

(4) Implications for educational practice.

The findings of this study have several implications for educational practice. Firstly, integrating chess into the school curriculum or as an extracurricular activity can be a useful supplement to traditional educational methods. Secondly, individualized instruction tailored to the needs of individual children (particularly those with learning disabilities or attention deficits) can maximizes the benefits of chess instruction. Finally, the results of the study suggest that parental involvement and home practice can further enhance the positive effects of chess instruction, highlighting the importance of a collaborative approach between school and home.

## 6 Conclusions and policy implications

This study confirmed the positive effects of chess teaching in the intellectual development of young children through systematic empirical analyses. The findings suggest that chess not only significantly enhances young children’s cognitive abilities, such as attention, memory, and logical thinking, but also plays an important role in emotional resilience (e.g., patience and self-discipline) as well as academic performance (e.g., math and reading scores). These enhancements are interrelated, suggesting that chess is a comprehensive educational tool that can promote the holistic development of young children.

Based on these findings, it is recommended that chess instruction be introduced into early childhood education programmed as a supplement to traditional educational methods. At the same time, professional training in chess teaching should be provided to teachers to help them master strategies and methods for integrating chess into the classroom. In addition, parents should be encouraged to participate in their children’s chess learning and consolidate their learning through home practice. Schools and the education sector should provide relevant resources and support to ensure the effective implementation of chess teaching. Individualized chess teaching programmed should be designed for children with special needs to meet the learning needs of different children. Finally, schools should be provided with the necessary resource support, including chess teaching aids, teaching materials and teacher training funds, to ensure the smooth implementation of chess teaching.

To provide a clearer understanding of the proposed educational strategy, Table 4 presents a summary of the key components and their interrelationship.

**Table 4:** Summary of the proposed educational strategy.

Component	Description
Chess teaching integration	Introduce chess instruction into early childhood education as a supplement to traditional methods
Teacher training	Provide professional training for teachers to effectively integrate chess into the classroom
Parental involvement	Encourage parents to participate in their children’s chess learning and support home practice
Resource support	Ensure schools have the necessary resources, including teaching aids, materials, and funding for teacher training
Individualized programs	Design individualized chess teaching programs for children with special needs
Holistic development	Promote the holistic development of young children by enhancing cognitive abilities, emotional resilience, and academic performance

While this study provides valuable insights into the application of chess teaching in early childhood education, it is important to acknowledge several limitations. One significant limitation is the potential lack of cultural generalizability. The study was conducted in a specific cultural context, and the findings may not be directly applicable to other cultural settings. Future research should explore the effectiveness of chess teaching in diverse cultural contexts to determine its broader applicability. Additionally, the study relied on a quasi-experimental design, which may limit the causal inferences that can be drawn. Future research should consider using randomized controlled trials to provide more robust evidence of the impact of chess teaching on children’s intellectual development. Furthermore, the study focused on short-term outcomes, and future research should include long-term follow-up studies to assess the sustained effects of chess instruction on children’s cognitive, emotional, and academic development.

In conclusion, chess teaching, as an effective educational tool, can significantly contribute to the intellectual development of young children. Educators, parents and policy makers should work together to incorporate chess into the early childhood education system to support the holistic development of young children.

## Data Availability

The original contributions presented in the study are included in the article/supplementary material, further inquiries can be directed to the corresponding author.
